# Chronic pain among homeless persons: characteristics, treatment, and barriers to management

**DOI:** 10.1186/1471-2296-12-73

**Published:** 2011-07-08

**Authors:** Stephen W Hwang, Emma Wilkins, Catharine Chambers, Eileen Estrabillo, Jon Berends, Anna MacDonald

**Affiliations:** 1 Centre for Research on Inner City Health, The Keenan Research Centre in the Li Ka Shing Knowledge Institute of St. Michael's Hospital, Toronto, Canada; 2 Division of General Internal Medicine, Department of Medicine, University of Toronto, Canada

## Abstract

**Background:**

Little information is available on the problem of chronic pain among homeless individuals. This study aimed to describe the characteristics of and treatments for chronic pain, barriers to pain management, concurrent medical conditions, and substance use among a representative sample of homeless single adult shelter users who experience chronic pain in Toronto, Canada.

**Methods:**

Participants were randomly selected at shelters for single homeless adults between September 2007 and February 2008 and screened for chronic pain, defined as having pain in the body for ≥ 3 months or receiving treatment for pain that started ≥ 3 months ago. Cross-sectional surveys obtained information on demographic characteristics, characteristics of and treatments for chronic pain, barriers to pain management, concurrent medical conditions, and substance use. Whenever possible, participants' physicians were also interviewed.

**Results:**

Among 152 homeless participants who experienced chronic pain, 11 (8%) were classified as Chronic Pain Grade I (low disability-low intensity), 47 (32%) as Grade II (low disability-high intensity), 34 (23%) as Grade III (high disability-moderately limiting), and 54 (37%) as Grade IV (high disability-severely limiting). The most common self-reported barriers to pain management were stress of shelter life, inability to afford prescription medications, and poor sleeping conditions. Participants reported using over-the-counter medications (48%), street drugs (46%), prescribed medications (43%), and alcohol (29%) to treat their pain. Of the 61 interviewed physicians, only 51% reported treating the patient's pain. The most common physician-reported difficulties with pain management were reluctance to prescribe narcotics due to the patient's history of substance abuse, psychiatric comorbidities, frequently missed appointments, and difficulty getting the patient to take medications correctly.

**Conclusions:**

Clinicians who provide healthcare for homeless people should screen for chronic pain and discuss barriers to effective pain management with their patients.

## Background

Chronic pain, defined as "pain that persists beyond normal tissue healing time, which is assumed to be 3 months" [1], is highly prevalent in the general population [2-6]. In the United States, 11% of the general population suffers from chronic pain [7]. In a Canadian survey, 25% of respondents reported continuous or intermittent pain lasting 6 months or more [3]. Chronic pain has a major adverse impact on quality of life and is a frequent reason for health care visits, yet clinicians often express a lack of comfort in treating patients with chronic pain [8,9].

Homelessness is a serious issue across North America: in the United States, about 3.5 million adults and children experience homelessness every year, and over 800,000 individuals are homeless at any one time [10]. In Canada, an estimated 150,000 to 300,000 people experience homelessness each year [11], and in Toronto, Ontario, about 5,000 individuals are homeless on any given night [12]. Chronic pain is thought to be common among homeless people, in part due to frequent injuries and the high prevalence of concurrent health conditions [13]. The management of chronic pain may be particularly challenging in this population because of barriers to obtaining health care, comorbid substance use and mental illness, and lack of a stable living environment [13,14]. However, few published studies have examined the problem of chronic pain among homeless individuals.

The purpose of this study was to describe the characteristics of and treatments for chronic pain, barriers to pain management, concurrent medical conditions, and substance use among a representative sample of homeless single adult shelter users who experience chronic pain in Toronto, Canada. We sought to determine whether these characteristics varied according to the participants' Chronic Pain Grade, a validated measure of overall pain severity based on the patients' intensity, disability, and duration of chronic pain [15,16], and hypothesized that participants who have more severe chronic pain would be more likely to require treatment for pain management, experience a greater number of barriers to pain management, have more concurrent medical conditions, and would report more frequent substance use. Whenever possible, data were also obtained from the patients' regular physicians or a physician whom the patient had seen for pain in the past three months. These data provided information on the patients' chronic pain history, treatments being prescribed or recommended for managing the patients' pain, and problems experienced in managing the patients' pain.

## Methods

### Sampling Design

Study participants aged 18 years or older were recruited between September 2007 and February 2008 at 17 shelters for single homeless adults (i.e., adults who do not live with a partner or dependent children) in Toronto. Ten were shelters for men only, five for women only, and two for men and women. At each shelter, individuals were selected from the shelter's bed list using a random number generator. If a selected individual could not be located, was unwilling to participate, or was not eligible, another individual was randomly selected. The target number of participants at each shelter was proportionate to the number of shelter beds. A total of 150 participants with chronic pain were chosen to provide a 95% confidence interval of ± 8% for the prevalence of uncontrolled pain.

Individuals were screened based on three questions: "Do you have pain in your body?"; "Are you being treated for pain in your body?"; and "How long ago did your pain start?" Individuals were eligible to participate if they had experienced pain for ≥ 3 months, regardless of whether they were receiving treatment for their pain. The pain could be either intermittent or continuous over time and either regional or widespread. All study participants provided written informed consent. Participants received $10 for completing the survey. This study was approved by the Research Ethics Board at St. Michael's Hospital.

### Survey Instrument

Data were obtained from a cross-sectional survey on demographic characteristics, history of homelessness, chronic pain history, pain management treatments, barriers to pain management, concurrent medical conditions, and substance use. The Chronic Pain Grade questionnaire was used to classify participants according to their overall pain severity [15,16]. This previously validated seven-item instrument measures chronic pain severity in three dimensions: intensity, disability, and duration. Participants were asked to rate pain intensity (current pain, worst pain in past six months, average pain in past six months) and pain-related disability (daily activities, social activities, and work activities) in the past six months on a 0-10 point scale and report on their duration of pain-related disability in the past six months. The instrument generates an overall score, which corresponds to qualitative differences in pain severity: Grade I (low disability-low intensity), Grade II (low disability-high intensity), Grade III (high disability-moderately limiting), and Grade IV (high disability-severely limiting).

Information was obtained on the participants' chronic pain history, including duration, location, and cause of pain. Participants were asked about their use of treatments for their pain in the past three months: over-the-counter medications, prescription medications, alcohol, street drugs, and other therapies (including herbal remedies, acupuncture, physical therapy, psychotherapy, massage therapy, and relaxation techniques).

To examine barriers to chronic pain management, participants were asked, "Can you name any problems you have had in managing your pain?" If participants had difficulty understanding this question, they were asked "Do you find it difficult to take care of your pain?" and if the response was affirmative, they were subsequently asked "What makes it difficult for you to take care of your pain?" Participants' spontaneous responses were recorded verbatim and categorized according to the following categories: health care system (general and physician-related), shelter system, biomedical, financial, personal, and physical environment. Participants' responses were also prompted using a list of common barriers previously identified through pilot testing. Participants were asked to report on their perceived level of pain control on a four-point scale (very uncontrolled to very controlled) and level of satisfaction with medical care on a four-point scale (very unsatisfied to very satisfied).

Concurrent medical conditions were assessed using self-report items. Participants were asked to report alcohol and drug use, excluding prescription medications taken on the advice of a doctor or nurse, in the past three months. For each substance used, participants were asked to report frequency of use and the perceived effect of the drug on their pain. The CAGE questionnaire was administered to participants who reported any alcohol use in the past three months in order to assess alcohol dependency [17].

### Physician Interviews

Participants were asked to consent to interview their physician about health conditions and pain management. These physicians included any family doctor or specialist whom the participant was seeing regularly or had seen for pain in the past three months. If a participant reported having more than one physician, the physician who was treating or aware of the chronic pain was selected. A minimum of four attempts were made to conduct a telephone interview with each physician.

If physicians were aware of their patient's chronic pain, they were asked about the duration, location, and cause of the pain; the medications or treatments being provided for pain; the problems experienced in managing their patient's pain; and the problems experienced by the patient in managing their own pain. The physicians, who were not informed that their patient was homeless at the time of the study, were also asked to describe their understanding of the patient's housing situation.

### Data Analysis

Bivariate analyses were conducted to assess the association between Chronic Pain Grade and demographic characteristics, history of homelessness, chronic pain history, pain management treatments, barriers to pain management, concurrent medical conditions, and substance use. Comparisons were made across Chronic Pain Grades (I, II, III, and IV) using one-way Analysis of Variance (ANOVA) for continuous variables and chi-square tests for categorical variables. Comparisons were also made between participants who had corresponding physician data and those who did not using ANOVA for continuous variables and chi-square tests for categorical variables. All analyses were performed using SPSS 15.0 for Windows (SPSS Inc., Chicago, IL) and STATA 9.0 (StataCorp LP, College Station, TX).

## Results

### Participant Survey

Of 312 shelter residents who were screened for eligibility, 162 had chronic pain according to our study definition; 10 individuals with chronic pain refused to participate, resulting in a final sample of 152 participants (94% of those eligible) who were enrolled in the study. On average, participants reported that they had experienced chronic pain for 10.3 years (95% CI: 8.7-11.9 years); the average age of onset was 35.8 years old (95% CI: 33.7-38.0 years old). Chronic Pain Grade scores were missing for 6 participants. Among the remaining participants, 11 (7.5%) were classified as Chronic Pain Grade I (low disability-low intensity), 47 (32.2%) as Grade II (low disability-high intensity), 34 (23.3%) as Grade III (high disability-moderately limiting), and 54 (37.0%) as Grade IV (high disability-severely limiting).

Demographic information, characteristics of and treatments used for pain, chronic medical conditions, and substance use according to Chronic Pain Grade are shown in Table 1. The three most common locations of pain were back (52.0%), knees (28.9%), and shoulders (21.1%). Participants classified as having more severe pain (as indicated by Chronic Pain Grade) were more likely to report that the location of their pain was their back (Χ^2 ^= 11.7, df = 3, p = 0.009) or their legs (Χ^2 ^= 11.6, df = 3, p = 0.009). Injuries were the most common cause of participants' chronic pain.

**Table 1:** Demographic characteristics and chronic pain history among homeless participants by Chronic Pain Grade (n = 146)

Characteristic	Chronic Pain				p-value
		
	Grade I (n = 11)	Grade II (n = 47)	Grade III (n = 34)	Grade IV (n = 54)	
Age (years), mean (sd)	48.2 (9.2)	44.9 (11.7)	45.9 (12.6)	46.2 (9.3)	0.826
Sex, n (%)					0.378*
Male	7 (63.6)	39 (83.0)	29 (85.3)	43 (79.6)	
Female	4 (36.4)	7 (14.9)	5 (14.7)	11 (20.4)	
Transgendered	0 (0.0)	1 (2.1)	0 (0.0)	0 (0.0)	
Lifetime duration of homelessness, n (%)					0.665
< 2 years	5 (45.5)	25 (53.2)	16 (47.1)	22 (40.7)	
≥ 2 years	6 (54.5)	22 (46.8)	18 (52.9)	32 (59.3)	
Source of income, n (%)^†^					
Disability benefits	2 (18.2)	10 (21.3)	11 (33.3)	15 (27.8)	0.595
Social assistance	3 (27.3)	9 (19.1)	8 (24.2)	11 (20.4)	0.906
Wages or salary	4 (36.4)	11 (23.4)	3 (9.1)	4 (7.4)	0.020
Highest level of education, n (%)					0.084
Less than high school	1 (9.1)	18 (38.3)	15 (44.1)	27 (50.0)	
High school or equivalent	10 (90.9)	29 (61.7)	19 (55.9)	27 (50.0)	
Has a regular medical doctor, n (%)	7 (63.6)	20 (42.6)	21 (61.8)	36 (66.7)	0.087
Duration of chronic pain (years), mean (sd)^‡^	7.5 (6.8)	9.8 (9.8)	8.3 (9.0)	12.5 (10.9)	0.184
Age at onset of pain (years), mean (sd)	40.6 (11.9)	35.4 (13.6)	37.6 (13.3)	33.7 (13.2)	0.339
Location of pain, n (%)^†^					
Back	2 (18.2)	18 (38.3)	20 (58.8)	34 (63.0)	0.009
Knees	4 (36.4)	11 (23.4)	8 (23.5)	18 (33.3)	0.579
Shoulders	2 (18.2)	7 (14.9)	6 (17.6)	16 (29.6)	0.292
Feet	2 (18.2)	11 (23.4)	5 (14.7)	9 (16.7)	0.754
Legs	0 (0.0)	5 (10.6)	3 (8.8)	16 (29.6)	0.009
Hands	0 (0.0)	7 (14.9)	5 (14.7)	10 (18.5)	0.483
Hips	1 (9.1)	7 (14.9)	5 (14.7)	8 (14.8)	0.965
Ankles	3 (27.3)	6 (12.8)	2 (5.9)	10 (18.5)	0.228
Abdomen/genitourinary	1 (9.1)	7 (14.9)	2 (5.9)	8 (14.8)	0.571
Neck	1 (9.1)	5 (10.6)	2 (5.9)	8 (14.8)	0.623
Widespread pain	0 (0.0)	1 (2.1)	0 (0.0)	3 (5.6)	--
Other location	5 (45.5)	13 (27.7)	9 (26.5)	28 (51.9)	--
Cause of pain, n (%)^†^					
Injury	7 (63.6)	26 (55.3)	20 (58.8)	34 (63.0)	0.875
Arthritis	1 (9.1)	5 (10.6)	7 (20.6)	13 (24.1)	0.278
Other	2 (18.2)	21 (44.7)	15 (44.1)	23 (42.6)	--
Don't know	1 (9.1)	7 (14.9)	6 (17.6)	11 (20.4)	0.784
Unmet needs for pain management, n (%)	2 (18.2)	12 (25.5)	9 (26.5)	16 (29.6)	0.878
Number of barriers to pain management, mean (sd)^§^	2.6 (2.7)	3.7 (3.2)	5.6 (3.1)	7.3 (3.8)	< 0.001
Pain control, n (%)					< 0.001
Very or somewhat controlled	10 (90.9)	35 (76.1)	14 (41.2)	19 (35.2)	
Very or somewhat uncontrolled	1 (9.1)	11 (23.9)	20 (58.8)	35 (64.8)	
Satisfaction with medical care, n (%)**					0.049
Very or somewhat satisfied	4 (100.0)	14 (63.6)	16 (72.7)	16 (44.4)	
Very or somewhat unsatisfied	0 (0.0)	8 (36.4)	6 (27.3)	20 (55.6)	
Currently being treated for pain in body, n (%)	1 (9.1)	16 (34.0)	18 (52.9)	25 (46.3)	0.042
Treatments used for pain in past 3 months, n (%)^†^					
OTC medications	5 (45.5)	21 (44.7)	19 (55.9)	25 (46.3)	0.766
Street drugs	3 (27.3)	18 (38.3)	18 (52.9)	28 (51.9)	0.253
Prescription medications^††^	1 (9.1)	13 (27.7)	21 (61.8)	30 (55.6)	< 0.001
Alcohol	1 (9.1)	15 (31.9)	9 (26.5)	18 (33.3)	0.412
Other therapies^‡‡^	3 (27.3)	14 (29.8)	4 (11.8)	14 (25.9)	--
Concurrent medical conditions^†^					
Depression	4 (36.4)	15 (31.9)	16 (47.1)	23 (42.6)	0.532
Arthritis	3 (27.3)	12 (25.5)	11 (32.4)	26 (48.1)	0.099
Asthma	1 (9.1)	11 (23.4)	15 (44.1)	18 (33.3)	0.085
Liver problems or hepatitis	2 (18.2)	11 (23.4)	10 (29.4)	19 (35.2)	0.502
High blood pressure	3 (27.3)	9 (19.1)	10 (29.4)	18 (33.3)	0.452
Migraine headaches	0 (0.0)	7 (14.9)	12 (35.3)	19 (35.2)	0.013
COPD	1 (9.1)	7 (14.9)	7 (20.6)	14 (25.9)	0.425
Stomach or intestinal ulcers	0 (0.0)	5 (10.6)	5 (14.7)	14 (25.9)	0.074
Heart disease	0 (0.0)	2 (4.3)	7 (20.6)	8 (14.8)	0.068
Other conditions	4 (36.4)	16 (34.0)	20 (58.8)	36 (66.7)	--
Count of concurrent medical conditions, mean (sd)	1.6 (0.9)	2.2 (1.8)	3.5 (2.1)	4.0 (2.7)	< 0.001
Substance use in past 3 months, n (%)^† §§^					
Alcohol	5 (45.5)	31 (66.0)	21 (61.8)	36 (66.7)	0.583
Marijuana	2 (18.2)	23 (48.9)	21 (61.8)	26 (48.1)	0.093
Cocaine and other stimulants	3 (27.3)	14 (29.8)	18 (52.9)	17 (31.5)	0.117
Opiates***	3 (27.3)	16 (34.0)	16 (47.1)	17 (31.5)	0.435
Sleeping pills or sedatives	2 (18.2)	8 (17.0)	7 (20.6)	10 (18.5)	0.983
Other drugs	0 (0.0)	2 (4.3)	0 (0.0)	3 (5.6)	--
CAGE score, mean (sd)^†††^	0.6 (0.9)	1.2 (1.4)	1.3 (1.5)	1.6 (1.6)	0.500

OTC = over the counter; COPD = chronic obstructive pulmonary disease (incl. chronic bronchitis and emphysema);

-- = statistical test not performed

Notes:

*Chi-square test excludes transgendered participant

^† ^Participants could select more than one option; percentages may not add to 100%

^‡ ^In order to be eligible for the study, participates had to have pain lasting at least three months

^§ ^Includes both spontaneous responses and those that were prompted

**Among participants who are receiving medical care

^†† ^Must have been prescribed by physician

^‡‡ ^Includes herbal remedies, acupuncture, physical therapy, psychotherapy, massage therapy, and relaxation techniques

^§§ ^Not prescribed by physician

***Includes Tylenol 2 or 3 containing codeine, oxycodone, morphine, heroin, methadone, and other opiates

^††† ^Among participants who have used alcohol in past three months.

**Figure 1:**
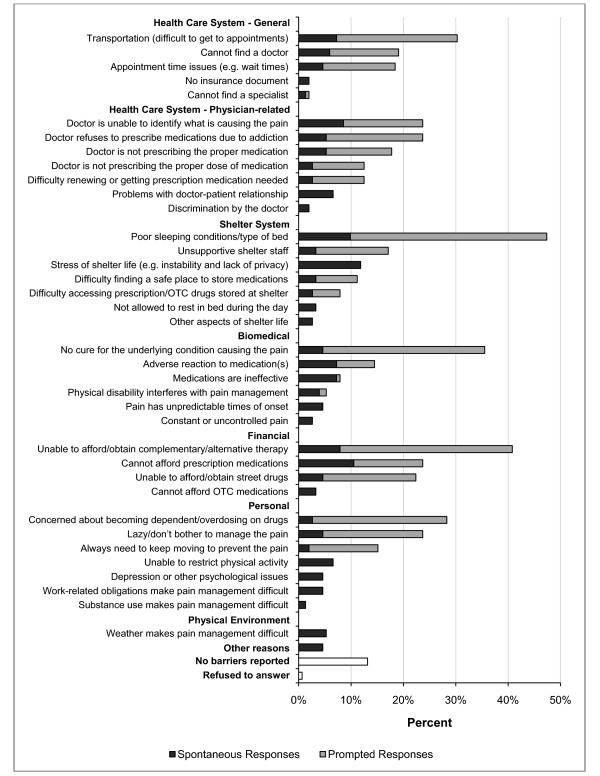
**Self-reported barriers to pain management, spontaneous and prompted responses**.

Barriers to chronic pain management are shown in Figure 1. The stress of shelter life (e.g. instability and lack of privacy), inability to afford prescription medications, poor sleeping conditions, inability of physicians to identify the cause of pain, inability to afford (or obtain) complementary and alternative therapies, transportation issues (e.g. difficulties getting to medical appointments), adverse reactions to medications, belief that the medications are ineffective, problems with the doctor-patient relationship (other than discrimination), and inability to restrict physical activities were all listed as common barriers. Participants with more severe pain were more likely to report a higher number of barriers to pain management (F = 10.6, df = 3, 142, p < 0.001).

Eighty-five participants (55.9%) reported having a regular medical doctor; of these, 60 (70.6%) reported that this physician was treating their chronic pain. Fifty-one participants (33.6%) had sought care for pain from a physician other than their regular medical doctor in the past three months. Forty participants (26.5%) reported that they had needed health care for their pain in the past three months but had not been able to get it. Of those receiving medical care for pain (n = 88), 58.0% were "very" or "somewhat" satisfied with their care. Chronic pain severity was significantly and inversely associated with participants reporting they were "very" or "somewhat" satisfied with their medical care (Χ^2 ^= 25.0, df = 3, p < 0.001) and was marginally and inversely associated with participants reported that their pain is "very" or "somewhat" controlled (Χ^2 ^= 7.9, df = 3, p = 0.049).

Less than half of participants (41.4%) were currently being treated for their pain. Treatments included over-the-counter medications (48.0%), street drugs (46.1%), medications prescribed by a physician (43.4%), alcohol (28.9%), and other treatments (including herbal remedies, acupuncture, physical therapy, psychotherapy, massage therapy, and relaxation techniques) (23.7%). Thirty participants (19.7%) reported concurrent use of both street drugs and medications prescribed by a physician, and 39 (25.7%) reported concurrent use street drugs and over-the-counter medications. Participants with more severe pain were more likely to report using medications prescribed by a physician to treat their pain (Χ^2 ^= 17.8, df = 3, p < 0.001). The number of self-reported concurrent medical conditions increased with increasing chronic pain severity (F = 7.7, df = 3, 142, p < 0.001).

**Figure 2:**
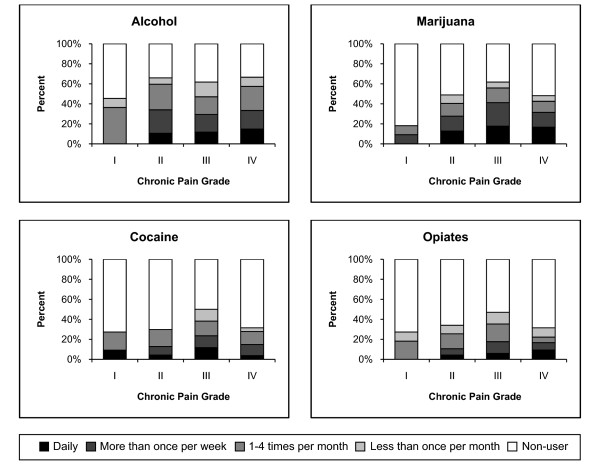
**Drug use frequency among homeless participants by Chronic Pain Grade (n = 146)**.

Participants reported using the following regulated and illicit substances in the past three months: alcohol (63.6%), marijuana (49.7%), cocaine and other stimulants (35.1%), opiates (including codeine, oxycodone, morphine, methadone, and heroin) (35.1%), and sleeping pills or sedatives (17.9%). CAGE scores, which measure alcohol dependency, did not significantly differ by chronic pain severity (Table 1). Frequency of drug use by pain severity is shown in Figure 2.

### Physician Survey

Of 152 participants, 130 provided the name of at least one treating physician and gave consent for their physician to be contacted. However, a number of these physicians could not be located, declined to participate, had no record of the patient, had not seen the patient within our specified time frame (within the last 12 months for primary care providers and within the last three months for other physicians), or had seen the patient on only one occasion. Thus, interviews were completed with 61 physicians, representing 40% of study participants. Participants with physician data were significantly older (50.0 vs. 43.5 years old; F = 13.9, df = 1, 150, p < 0.001) and had more severe chronic pain (45.5% vs. 31.9% classified as Grade IV; Χ^2 ^= 8.0, df = 3, p = 0.046) compared to those without physician data. Not surprisingly, participants with physician data were also were more likely to receive disability benefits (40.0% vs. 17.8%; Χ^2 ^= 9.1, df = 1, p = 0.003) and were more likely to report currently being treated for pain (59.0% vs. 29.7%; Χ^2 ^= 13.0, df = 3, p < 0.001). The two groups did not significantly differ with respect to sex, lifetime duration of homelessness, other sources of income, education, or duration of chronic pain.

Only 39 (63.9%) of the 61 physicians contacted were aware of their patient's chronic pain, and only 31 (50.8%) were treating their patient's pain. Among the physicians who were treating the study participant's pain, 16 (51.6%) were using a special prescribing practice (e.g., dispensing only a two-week, one-day, or single dose supply of medication at one time), 13 (41.9%) specifically stated that treatment with opiates should be avoided in their patient, and 24 (77.4%) reported difficulties managing their patient's pain. The difficulties most commonly reported were a reluctance to prescribe narcotics due to a history of substance abuse in the patient; treatment challenges due to psychiatric comorbidities; frequently missed appointments; difficulty getting the patient to take medications correctly; and the patient's lack of coverage for alternative or complementary therapies. When asked to describe their patient's housing situation, 17 (27.9%) physicians were not aware of their patient's current or past history of homelessness.

## Discussion

In this study of homeless shelter residents in Toronto, more than one-third of study participants were classified as Chronic Pain Grade IV, indicative of high intensity and high disability. Almost half the participants reported use of street drugs to treat their pain, and 29% reported use of alcohol. Among participants' physicians who were interviewed, only half were treating the participant's pain, and 77% of treating physicians reported difficulties managing their patient's pain due to factors such as a history of addiction in the patient, psychiatric comorbidities, and missed appointments.

Homeless individuals reported numerous barriers to chronic pain management, and reporting a larger number of barriers was significantly associated with increasing pain severity. Commonly reported barriers included the stress of shelter life, inability to afford prescription medications, and poor sleeping conditions. While this study was conducted in a health care system that provides universal health insurance, these self-reported barriers are similar to those described by clinicians working with homeless individuals in the United States, where lack of health insurance poses an additional barrier to obtaining needed care [10,13].

To our knowledge, this is the first study in the peer-reviewed literature to describe the severity and management of chronic pain in a homeless population and to examine both homeless patients' and their physicians' perspectives on chronic pain management. The study enrolled a random sample of shelter users with chronic pain in a major North American city and used validated instruments to assess chronic pain severity. However, despite these strengths, certain limitations should be noted. Our study was designed to obtain a representative sample of homeless individuals with chronic pain and was not intended to generate an estimate of the prevalence of chronic pain among homeless people. Our sample was restricted to homeless single adults who used shelters; consequently, our results are not generalizable to homeless families or youth. While our sampling strategy excluded homeless individuals who do not use shelters, the size of this subgroup is minimal in the city of Toronto, with only 8% of Toronto's homeless population individuals staying outdoors on any given night [12]. Chronic pain was determined using self-report, and some individuals may have falsely reported the presence of chronic pain in order to participate. In order to reduce the likelihood of this occurrence, the study's focus and eligibility criteria were not disclosed to individuals who were being screened. Finally, we were able to complete a physician interview for only 40% of study participants, in part due to the lack of a regular source of care for many homeless individuals.

## Conclusions

This study's findings demonstrate a need for improved approaches to the management of chronic pain in the homeless population. Outreach efforts may be necessary to engage homeless individuals who suffer from chronic pain but are not seeking appropriate care. Health care providers who work with marginalized populations should familiarize themselves with their patients' housing situations and routinely screen individuals who are homeless for chronic pain. Clinicians should inquire about barriers to pain management, such as financial ability to obtain appropriate over-the-counter and prescription medications and the adverse effects of homeless people's living and sleeping conditions. This latter issue raises the question of whether interventions that provide housing for homeless individuals might also have substantial positive effects on pain control.

Finally, the physician interviews suggest that the management of chronic pain in homeless individuals who have a history of substance use dependency poses serious challenges to clinicians, who are often understandably concerned about the potential abuse of opiate analgesics [18,19]. These concerns, while not unjustified, should not be considered a reason to avoid addressing chronic pain management for these patients. Further research is needed to evaluate the effectiveness of carefully specified pain management protocols, including pharmacologic and non-pharmacologic therapies, for the control of chronic pain in this population.

## Competing interests

The authors declare that they have no competing interests.

## Authors' contributions

SWH, AM, and EW contributed to the study concept and design. EW, EE, JB, and AM acquired the data. SWH, EW, and CC performed the analyses, interpreted the data, and drafted and revised the manuscript. EE, JB, and AM critically revised the manuscript for important intellectual content. All of the authors approved the final manuscript.

## Pre-publication history

The pre-publication history for this paper can be accessed here:

http://www.biomedcentral.com/1471-2296/12/73/prepub
